# The Use of Chemical Flocculants and Chitosan as a Pre-Concentration Step in the Harvesting Process of Three Native Microalgae Species from the Canary Islands Cultivated Outdoors at the Pilot Scale

**DOI:** 10.3390/microorganisms12122583

**Published:** 2024-12-13

**Authors:** Laura Figueira Garcia, Zivan Gojkovic, Marianna Venuleo, Flavio Guidi, Eduardo Portillo

**Affiliations:** Instituto Tecnológico de Canarias (ITC), Playa de Pozo Izquierdo, s/n, 35119 Santa Lucía de Tirajana, Gran Canaria, Spain

**Keywords:** microalgae harvesting, chemical flocculants, chitosan, pilot-scale flocculation

## Abstract

Biomass harvesting represents one of the main bottlenecks in microalgae large-scale production. Solid–liquid separation of the biomass accounts for 30% of the total production costs, which can be reduced by the use of flocculants as a pre-concentration step in the downstream process. The natural polymer chitosan and the two chemical flocculants FeCl_3_ and AlCl_3_ were tested on freshwater *Chlorella sorokiniana* and two marine algae, *Dunaliella tertiolecta* and *Tetraselmis striata*. A preliminary screening at the laboratory scale was performed to detect the most suitable doses of flocculants. On the basis of these results, selected doses were tested on the pilot scale, using the flocculants for a pre-concentration step and the centrifugation as a second step to confirm the effectiveness of flocculants in a realistic operational environment. The biomass recoveries (R_pilot_, %) of 100 L cultures were as follows: (1) for *T. striata*, R_pilot_ = 94.6% for 0.08 g/L AlCl_3_, 88.4% for 0.1 g/L FeCl_3_, and 68.3% for 0.04 g/L chitosan; (2) for *D. tertiolecta*, R_pilot_ = 81.7% for 0.1 g/L AlCl_3_, 87.9% for 0.2 g/L FeCl_3_, and 81.6% for 0.1 g/L chitosan; and (3) for *C. sorokiniana*, R_pilot_ = 89.6% for 0.1 g/L AlCl_3_, 98.6% for 0.2 g/L FeCl_3_, and 68.3% for 0.1 g/L chitosan. Flocculation reduced the harvesting costs by 85.9 ± 4.5% using chemical flocculants. Excesses of aluminum and iron in the biomass could be solved by decreasing the pH in the biomass combined with washing. This is the first study, to the best of our knowledge, that investigates the pilot-scale flocculation of three native Canarian microalgal strains. A pilot-scale pre-concentration step before centrifugation can improve the yield and reduce costs in the microalgae harvesting process.

## 1. Introduction

Interest in microalgae biotechnology has increased in the last few decades due to its potential applications in aquaculture, the production of bioactive compounds (such as pigments), wastewater treatment, human consumption, the food supplement industry, and biofuel production [1,2,3,4,5,6]. One of the main bottlenecks in microalgae production is harvesting, which contributes to 30% of the total biomass production costs in open cultivation systems due to microalgal characteristics such as small cell sizes (1–30 µm in diameter), their colloidal stability in suspension, and their low concentrations in liquid culture medium (0.02–0.5% dry solids) [7,8,9,10,11]. The energy needed to harvest microalgae culture at 0.3 g_DW_/L by centrifugation was estimated to be 13.8 MJ/kg_DW_ [12], accounting for almost 75% of the combustion enthalpy of the dry microalgal biomass (approximately 20.0 MJ/kg_DW_) [13]. Thus, to achieve economically and environmentally friendly microalgae production processes, it is necessary to search for alternative low-energy and cost-effective harvesting techniques [14]. Large-scale microalgal harvesting techniques include filtration, flotation, gravity sedimentation, and centrifugation [8,9,12]. Flocculation is widely used to separate colloidal substances in water treatment plants and foods and beverages, and it has recently received significant scientific attention in the microalgae harvesting process [14] due to its simple and relatively low-cost operation. Its efficiency depends on several parameters including culture concentration and surface properties, pH and ionic strength of the culture medium, the flocculant type and dosage, and flocculation time [15,16,17,18]. There are several types of flocculation processes: physical, biological, and chemical. Flocculant agents can be divided into inorganic (aluminum, zinc, iron, and other cationic salts) and organic as a natural cationic polymer (chitosan, derived from crustacean shells) [14]. Inorganic flocculants act by rapidly neutralizing the negative charge on the microalgal cell surface, while organic flocculants form physical linkages with microalgal cells through patching, bridging, and sweeping [14]. Specifically, when chitosan is dissolved in an acid solution, a large number of amino groups in the molecule are protonated, and the positive amine ions neutralize the negative charge of microalgal cells inducing algal sedimentation by the adsorption bridging of the polymer chain [14].

Inorganic flocculants are generally cheap and widely used in water treatment applications with high efficiency. When applied to microalgal cultures, however, these flocculants may affect the quality of the biomass for its use in food, feed, and cosmetics due to the accumulation of metal salts and also complicate the reuse of the culture medium [10,19].

On the other hand, natural polymers such as chitosan, despite being more expensive inorganic flocculants, are more compatible with microalgal applications due to their nature: non-toxic, non-corrosive, safe to handle, biodegradable, and biocompatible [20]. Although it is less efficient than some synthetic polycationic polymers [7,21,22,23,24], it is a completely natural and non-toxic chemical product that allows further application of the biomass in products that are destined for human and animal nutrition or pharmaceutical applications. Furthermore, its complete biodegradability presents no additional burden to the environment. It is also easy to obtain chitosan in large quantities via general suppliers without the need for special permissions, and its bulk price is acceptable for large-scale biomass production [20,25]. Flocculation using different chemical or biological flocculants was tested on various microalgal species, including *Chlorella vulgaris* [26,27,28,29], *Chlorella sorokiniana* [25], *Chlorella minutissima* [30], *Dunaliella salina* [31], *Thalasiosira pseudonana* [23], *Phaeodactylum tricornutum* [32], *Neochloris oleoabundans* [29,33], *Nannochloropsis* sp. [34], *Scotelliopsis reticulata* [27], *Scenedesmus obliquus* [29,35], *Scenedesmus* sp. [36], *Tetraselmis suecica* [23], *Botryococcus braunii* [37], and *Microcystis aeruginosa* [38,39]. Flocculation has been widely investigated with different microalgae strains and flocculant types at the laboratory scale, but pilot-scale studies in an operational environment or an actual industrial facility are less common [23,26,31].

The aim of this study was to investigate pilot-scale flocculation as the pre-concentration step prior to centrifugation of different microalgal cultures. Based on the obtained results, the two-step harvesting process could be applied to large-scale cultures, which would greatly reduce the culture volumes for centrifugation and the costs of harvesting. This is the first study, to the best of our knowledge, that investigates the pilot-scale flocculation of three native Canarian microalgal strains using two chemical and one bio-flocculant.

## 2. Materials and Methods

### 2.1. Microalgae Strains and Indoor Culture Conditions

The marine microalga *Tetraselmis striata* BEA 1978B (GenBank access ID: MT012288) was isolated by the Spanish Bank of Algae (BEA) from the desalination plant (27°81′ N 15°42′ W) and then kept at the Canarian Institute of Technology (ITC, Gran Canaria, Spain) collection. Cultures were maintained in a modified Guillard’s seawater F/2 culture medium (with the addition of 1 mM of urea [40]), composed as follows: NaNO_3_, 150; CON_2_H_4_, 60; NaH_2_PO_4_·2H_2_O, 11.30; Na_2_EDTA, 4.16; FeCl_3_·6H_2_O, 3.15; CuSO_4_·5H_2_O, 0.01; ZnSO_4_·7H_2_O, 0.022; CoCl_2_·6H_2_O, 0.01; MnCl_2_·4H_2_O, 0.18; and Na_2_MoO_4_·2H_2_O, 0.006 (in mg/L) [41].

The marine microalga *Dunaliella tertiolecta* BEA 1976B (GenBank access ID: MT015966) was isolated at the desalination plant (27°81′ N 15°42′ W). Cultures for the indoor scaling up were maintained in Guillard’s seawater F/2 medium [42] composed as follows: NaNO_3_, 150; NaH_2_PO_4_·2H_2_O, 11.30; Na_2_EDTA, 4.16; FeCl_3_·6H_2_O, 3.15; CuSO_4_·5H_2_O, 0.01; ZnSO_4_·7H_2_O, 0.022; CoCl_2_·6H_2_O, 0.01; MnCl_2_· 4H_2_O, 0.18; and Na_2_MoO_4_·2H_2_O, 0.006 (in mg/L) [41].

The freshwater microalga *Chlorella sorokiniana* BEA 1922B was isolated by the Spanish Bank of Algae (BEA, Gran Canaria, Spain) from wastewater at a sewage treatment plant. *C. sorokiniana* was maintained in a modified BG11 growth medium [43] modified with 10% of natural seawater, composed as follows: NaNO_3_, 1500; K_2_HPO_4_, 120; urea, 15; FeSO_4_·7H_2_O, 6; citric acid, 10; MnCl_2_·4H_2_O, 1.5; ZnSO_4_·7H_2_O, 0.22; and CuSO_4_·5H_2_O, 0.025 (in mg/L).

Inoculums of the three strains were cultured in semi-continuous mode at the following laboratory conditions: light irradiation range between 200 and 300 µmol_photons_·m^−2^·s^−1^, pulse CO_2_ supply of 1% *v*/*v*, 1 L/min, and temperature of 25 ± 2 °C. They were grown in 250 mL flasks and then scaled up indoors to 8 L volume in polycarbonate vessels (Nalgene, Rochester (NY), USA) under the same experimental conditions to start the pilot-scale flocculation experiments.

### 2.2. Lab-Scale Flocculation

A laboratory screening with chemical flocculants (AlCl_3_ and FeCl_3_) and one natural polymer (high-molecular-weight chitosan) was performed to evaluate the minimum flocculant dose assuring a biomass recovery higher than 85% for each microalgal strain. All the flocculants were supplied by Sigma-Aldrich Co., St. Louis (MO), USA. The flocculant doses were set according to the following equation [30]:

(1) Coagulant_concentration (g/L)=0.2083 ·OD (750)

For the chemical flocculants, the chitosan doses were prepared based on the optimization process proposed by [20]. Flocculation experiments were performed with cultures of OD 750 = 0.8–1.0 [29] in the exponential phase by adding selected flocculants to an aliquot of 30 mL from the stock solution to form the desired final concentrations. The solutions were stirred with a vortex for 2 min, distributed in 4 mL cuvette trays in triplicates, and left to settle for 180 min. The optical density at 750 nm (OD 750) was measured in a UV/visible spectrophotometer (HACH Lange DR3900, Hach Lange GmbH, Düsseldorf, Germany) every 15 min. The lab-scale recovery (R_lab_, %) was calculated according to the following equation [29,44]:

(2) $$R_{\mathrm{lab}}\left(\%\right)=\frac{{\mathrm{OD}}_{750}\;\left(t_{0}\right)-{\;\mathrm{OD}}_{750}\;\left(t\right)}{{\mathrm{OD}}_{750}\;\left(t_{0}\right)}\cdot \;100$$

where OD_750_ (t_0_) is the optical density at the time zero, and OD_750_ (t) is the optical density of the culture at times 15, 30, 45, 60, 75, 90, 105, 120, 135, 150, 165, and 180 min.

### 2.3. Pilot-Scale Flocculation

Pilot-scale experiments were performed from May to August 2021 inside the 1500 m^2^ greenhouse covered by polycarbonate sheets at 3 m in height (Suntuf^®^ Plus, Palram Industries Ltd., Ramat Yohanan, Israel) at the ITC facilities in Pozo Izquierdo, Gran Canaria, Spain (27°49′ N, 15°25′ W), where excessive heating during the daytime was prevented by fan extractors. The ITC facilities were collated in a semi-desertic area, with dominant winds from the NNE, more than 10 h day length, temperatures ranging from 18 to 25 °C, and limited rainfall [45].

Cultures previously scaled up in the culture chamber from 250 mL Erlenmeyer flasks up to 8 L containers were inoculated in a 250 L raceway pond located in the greenhouse. The culture was completely transferred to a 1600 L raceway (as an inoculum), once it reached an OD 750 of 0.8–1.0. The culture was mixed by a paddlewheel at 20 rpm, and 1 L/min of pure CO_2_ was provided by an air diffuser to maintain the pH of the culture in a range of 6.0–8.0. Any eventual biological contaminants were successfully controlled by adding 1 mM of urea to the culture volume, as described by [40].

Cultures were monitored daily by measuring the pH with a pH-meter (Crison pH25+, Crison Instruments, Barcelona, Spain), the salinity and temperature with a salinometer equipped with a thermometer (inoLab WTW Cond-level 1, Xylem Analytics, Washington, DC, USA), and the maximum quantum yield (Qy) using a portable pulse amplitude-modulation (PAM) fluorimeter (AquaPen AP-100, Photon Systems Instruments, Brno, Czech Republic) and were tested under a light microscope (Leica DMi1, Leica Microsystems, Wetzlar, Germany, magnification ×40) for eventual biological contaminations. The culture growth was both assessed by optical density measurements spectrophotometrically at 750 nm and verified by dry weight measurements performed by the filtration of 10 mL of culture over pre-combusted and pre-weighted glass fiber filters (Whatman GC, Maidstone, Kent, United Kingdom), washed twice with 150 mL of ammonium formate (NH_4_HCO_2_) to remove salts [46], dried, and weighted. The biomass concentration, C_x_, was calculated based on the dried filter weight differences and expressed in g_DW_/L [47].

During all the flocculation experiments performed at the ambient temperature, the pH and salinity ranges were as follows: 6.4–7.1 and 35–42 g/L for *T. striata*, 7.1–7.8 and 37–39 g/L for *D. tertiolecta*, and 7.1–7.5 and 4.6–5.2 g/L for *C. sorokiniana*, respectively.

Once cultures reached an OD 750 of 0.8–1.0, a volume of 400 L from the 1600 L culture was evenly distributed to four 100 L column PBRs, of which 92 L was transferred directly to the column PBRs (2 m in height and 25 cm in diameter) with a peristaltic pump (Boyser AMP-22, Barcelona, Spain), and then the culture was kept at a constant aeration and mixed throughout the column by the injection of air provided by an air compressor (FIAC, Bologna, Italy). The doses of the flocculants (described above) were dissolved in the remaining 8 L of the culture and later added to the column PBRs to ensure the complete dissolution of the flocculants over the next 20 min, after which the air supply was stopped for 180 min for the biomass settlement.

For each microalga, three different doses of AlCl_3_, FeCl_3_, and chitosan were tested during three consecutive days, with one of the column PBRs containing the untreated microalgae culture as the control. The most effective dose of each flocculant, i.e., the minimum dose with the best trade-off between the centrifuge recovery and the recovery efficiency, was repeated at the last experimental day. The raceway of 1600 L was maintained in a semi-continuous mode during this process to ensure enough biomass and culture for the assays. Flocculation was considered successful when the recovery efficiency was >85%.

After 180 min, the two phases, the supernatant (containing the culture medium) and the concentrated phase (flocs), were separated. An aliquot of the supernatant was collected for measuring its OD 750, and the rest was discarded. Both settled and floating flocs of the treated cultures were harvested by desktop centrifuge, and an aliquot of the harvest was collected to measure the percentage of its water content. As the volume was very low due to the high efficiency of the flocculants, we used the desktop centrifuge (GEA Westfalia OTC3-02-137, GEA Westfalia Separator Group GmbH, Oelde, Germany). The culture was pumped with a peristaltic pump from the raceway to the centrifuge at a flow rate of 750 L/h. The centrifuge received a flow of 750 L/h and operated at 10,000 rpm. The biomass of the control culture had to be harvested in its entirety since the culture was homogenous, and no clear separation of phases occurred. The water content (W, %) was determined gravimetrically and calculated as the difference between the weight of a known amount of algal paste and the weight of the algal paste dried overnight at 105 °C in an oven (Carbolite-AX, Carbolite Gero, Hope, Derbyshire, United Kingdom).

The flocculation efficiency was calculated as follows:

(a) A single-step flocculation harvesting process was calculated as described above (Section 2.2), where the pilot-scale recovery equation is
  (3) $$R_{\mathrm{pilot}}\left(\%\right)=\frac{{\mathrm{OD}}_{750}\left(t_{0}\right)-{\;\mathrm{OD}}_{750}\left(\mathrm{supernatant}\_\mathrm{at}\_t_{180}\right)}{{\mathrm{OD}}_{750}\left(t_{0}\right)}\cdot 100$$
  where OD_750_ (t_0_) is the optical density of the culture before the addition of the flocculant and OD_750_ (supernatant_at_t_180_) is the optical density of the supernatant at the end of the flocculation experiment (at t = 180 min).
(b) Two-step flocculation harvesting is flocculation followed by a centrifugation process based on the calculation of the concentration factor (CF), centrifuge recovery (CR), and recovery efficiency (RE), as follows:
  (4) $$\mathrm{CF}=\frac{V_{0}}{V_{f}}$$
  where V_0_ is the initial culture volume of the PBRs (100 L) and V*_f_* is the volume of the flocs after the 180 min flocculation period.
  (5) $$\mathrm{CR}=\frac{C_{\mathrm{harvest}\;}-{\;C}_{\mathrm{flocculants}}}{V_{\mathrm{harvest}}}\cdot \frac{\left(1-\;W\right)}{{\mathrm{Cx}}_{0}}$$
  where C_harvest_ (g) is the weight of the paste harvested by the centrifuge, C_flocculants_ (g) is the weight of the flocculants added, V_harvest_ is the total harvested volume, and Cx_0_ is the biomass concentration of the control culture without the flocculants.
  (6) $$\mathrm{RE}\left(\%\right)=\frac{\mathrm{CR}\;\left(f\right)-\mathrm{CR}\left(0\right)}{\mathrm{CR}\;\left(f\right)}\cdot 100$$
  where CR (*f*) is the centrifuge recovery of the concentrated phase (flocs) and CR (0) is the centrifuge recovery of the untreated culture (control) at the end of the flocculation experiments.

### 2.4. Biomass Analysis

The concentrated phase of the treated cultures and the whole culture of the control were harvested from the PBRs at the end of the flocculation experiments. The biomass corresponding to the most effective flocculants for the three microalgae was analyzed in triplicate for its elemental composition.

The elemental composition, i.e., minerals, trace elements, and heavy metal content, was determined using inductively coupled plasma optical emission spectrometry ICP-OES (AVIO 500 Perkin Elmer, Waltham, Massachusetts, USA) after acid digestion of the biomass in Milestone Ethos Easy microwave digester (Milestone, Sorisole (BG), Italy).The water content was determined gravimetrically after drying the samples in an oven at 105 °C and cooling them down in a vacuum desiccator until the stabilization of the weight measurements. The ash content was determined gravimetrically by dry biomass combustion in a muffle furnace at 550 °C for 12 h.

### 2.5. Economic Viability Assessment

The technical specifications of the industrial centrifuge (RIERA-NADEU10, Granollers, Barcelona, Spain) that was used in our facility to harvest medium-volume raceways were applied to calculate the cost in EUR/m^3^ of the traditional harvesting method (centrifugation) and the two-step harvesting method (centrifugation of the flocs). The energy consumption (kWh/m^3^) was calculated by dividing the motor power (4 kW) and the centrifuge capacity (0.75 m^3^/h).

The cost of the centrifugation method Cost_cent_ (EUR/m^3^) was calculated by dividing the energy consumption and the price of the industrial electrical energy in the Canary Islands (0.22 EUR/kWh).

For the two-step harvesting method, the price was calculated as follows:

(7) $$\mathrm{Cost}\left(\mathrm{EUR}/m^{3}\right)=\frac{{\mathrm{Cos}t}_{cent}\left(\mathrm{EUR}/m^{3}\right)}{\mathrm{CF}}+\left[{\mathrm{Cos}t}_{f}\left(\mathrm{EUR}\right)\cdot F_{d}\left(g/m^{3}\right)\right]$$

where Cost*_f_* (EUR/g) is the price of the optimal dose of flocculant (F*_d_* (g/m^3^)) required to flocculate 1.0 m^3^ culture volume and CF is the concentration factor. All the flocculant prices were supplied by Spanish and European bulk suppliers, and the price range was in accordance with those already published in the studies [14,48].

### 2.6. Statistical Analysis

Statistical analyses were performed using Past4 software (Paleontological Statistics Software Package for Education and Data Analysis) [49].

Statistical differences between the aluminum, iron, ash, and water content were tested using Anderson–Darling tests for normality of the data; homogeneity of variance was tested by Levene’s test; and comparisons between groups were performed using the one-way ANOVA test followed by Tukey’s pairwise test to measure the significance degree at the 95% significance level (*p* < 0.05) for normally distributed data. When a normal distribution of the data could not be assumed, the Kruskal–Wallis test for equal medians followed by Dunn’s post hoc unpaired test were performed. The statistical significance was set to *p* < 0.05 for all the analyses.

## 3. Results

### 3.1. Evaluation of Flocculants in Laboratory Scale

**Figure 1:**
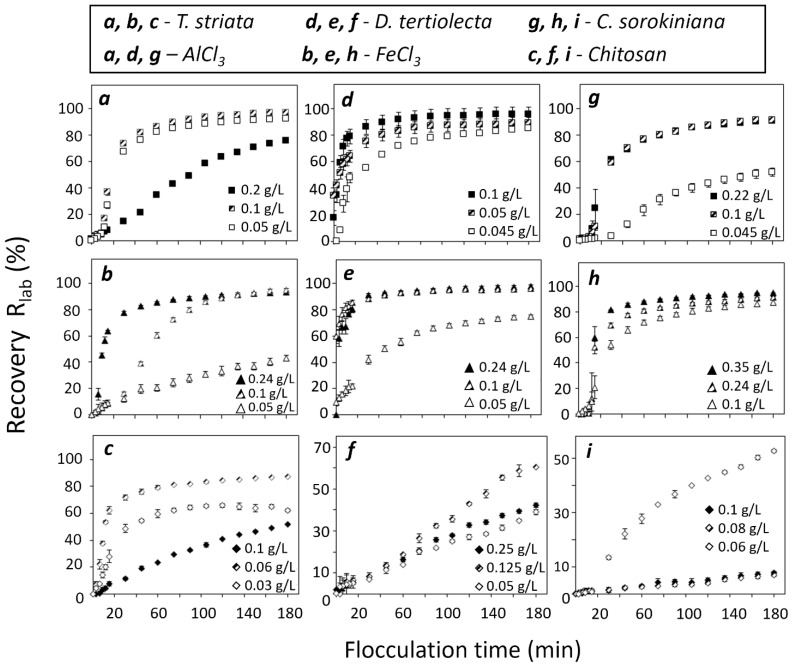
Recovery R_lab_ (%) of the selected species during the 180 min of flocculation. (**a**) Recovery (%) of *T. striata* added with AlCl_3_ at doses of 0.2 g/L, 0.1 g/L, and 0.05 g/L. (**b**) Recovery (%) of *T. striata* added with FeCl_3_ at doses of 0.24 g/L, 0.1 g/L, and 0.05 g/L. (**c**) Recovery (%) of *T. striata* added with chitosan at doses of 0.1 g/L, 0.06 g/L, and 0.03 g/L. (**d**) Recovery (%) of *D. tertiolecta* added with AlCl_3_ at doses of 0.1 g/L, 0.05 g/L, and 0.045 g/L. (**e**) Recovery (%) of *D. tertiolecta* added with FeCl_3_ at doses of 0.24 g/L, 0.1 g/L, and 0.05 g/L. (**f**) Recovery (%) of *D. tertiolecta* added with chitosan at doses of 0.25 g/L, 0.125g/L, and 0.05 g/L. (**g**) Recovery (%) of *C. sorokiniana* added with AlCl_3_ at doses of 0.22 g/L, 0.1 g/L, and 0.045 g/L. (**h**) Recovery (%) of *C. sorokiniana* added with FeCl_3_ at doses of 0.35 g/L, 0.24 g/L, and 0.1 g/L. (**i**) Recovery (%) of *C. sorokiniana* added with chitosan at doses of 0.1 g/L, 0.08 g/L, and 0.06 g/L. All data points represent the average of the triplicate measurements with corresponding standard deviation bars.

**Figure A1:**
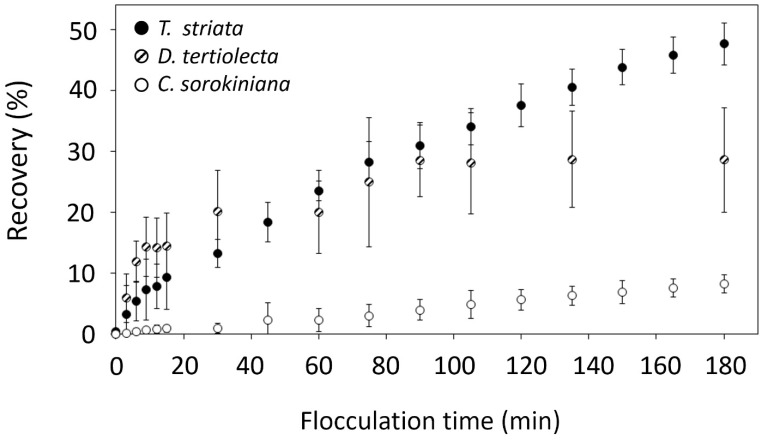
Recovery R_lab_ (%) of the three microalgae strains, *T. striata*, *D. tertiolecta*, and *C. sorokiniana*, subjected to gravity sedimentation for 180 min. All data points represent the average of the triplicate measurements with corresponding standard deviation bars.

For the three different strains of microalgae, different doses of AlCl_3_, FeCl_3_, and chitosan were tested to determine the minimum concentrations of the flocculants required to reach a recovery higher than 85% in a 180 min time period. The flocculant concentration range for each microalgae strain was selected according to the correlation equation between the cell concentration and the flocculant amount proposed by Papazi et al. (2010) [30]. The chitosan concentration range was selected based on the optimization process proposed by [20]. The best performing doses of the flocculation experiments are shown in Figure 1. To evaluate the auto-settling properties first, for the selected microalgae, the amount of biomass recovered after 180 min of gravity-induced, natural sedimentation was determined (Figure A1, Appendix A). For *Tetraselmis striata*, *Dunaliella tertiolecta*, and *Chlorella sorokiniana*, the final lab-scale recoveries (R_lab_ (%)) by gravity-induced sedimentation after 180 min of time were 47.6 ± 3.7%, 28.6 ± 8.5%, and 8.2 ± 1.5%, respectively (Figure A1, Appendix A).

The flocculation efficiency of AlCl_3_ in *T. striata* cultures was tested at concentrations of 0.2, 0.1, and 0.05 g/L (Figure 1a–c). After the addition of the flocculant, a clear interphase was observed after the first 20 min at all tested doses. Cultures with 0.1 and 0.05 g/L of AlCl_3_ reached the plateau (i.e., a recovery value that changed less than ±10% concerning the final recovery value) after 75 min, with R_lab_ values of 96.9 ± 0.2% and 92.2 ± 0.3%, respectively. Also, the higher (0.2 g/L) and lower (0.03 g/L) doses resulted in lower R_lab_ values (75.4 ± 0.9% and 71.6 ± 0.4%, respectively). The doses of FeCl_3_ applied were 0.24, 0.1, and 0.05 g/L. Unlike AlCl_3_, the R_lab_ obtained with FeCl_3_ was higher at lower doses (96.7 ± 0.5% at 0.08 g/L compared with 92.9 ± 0.5 at 0.2 g/L of FeCl_3_), with the lowest R_lab_ of 43.3 ± 2.1% obtained when 0.05 g/L of FeCl_3_ was applied. The doses of 0.2 and 0.08 g/L of ferric chloride had a similar trend, reaching the plateau after 60 and 75 min, respectively, while the recovery at a dose of 0.1 g/L was achieved faster after 45 min of reaction, reaching the plateau after 105 min of time. Although 0.24 g/L of FeCl_3_ flocculated the biomass faster, the recovery of the biomass was higher at a dose of 0.1 g/L FeCl_3_ (95.0% compared with 92.9%). Differently from AlCl_3_ and FeCl_3_, the maximum R_lab_ obtained when using chitosan (73.2 ± 1.1%) was achieved at the intermediate dose of 0.08 g/L (Figure 1c).

Regarding *D. tertiolecta*, AlCl_3_ was tested at doses of 0.1, 0.08, and 0.05 g/L, in which the R_lab_ increased with the increase in the flocculant dose, being the maximum (95.6 ± 0.2%) at 0.1 g/L (Figure 1d–f). FeCl_3_ was tested in doses of 0.2, 0.1, and 0.05 g/L, and similar to AlCl_3_, the maximum R_lab_ obtained (97.8 ± 1.1%) was at a dose of 0.2 g/L. Moreover, doses of 0.2 and 0.1 g/L of FeCl_3_ reached the plateau after 30 min of flocculation. Chitosan was more efficient at the intermediate concentration of 0.15 g/L with R_lab_ = 74.0 ± 1.2%, while the R_lab_ of the higher concentration (0.2 g/L) was 42.2 ± 0.7% (Figure 1f).

**Figure A2:**
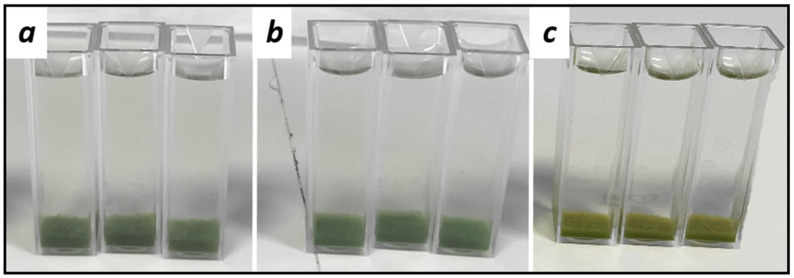
Comparison of the laboratory-scale flocculation results and color change with different concentrations of FeCl_3_: (**a**) 0.1 g/L FeCl_3_, (**b**) 0.24 g/L FeCl_3_, and (**c**) 0.35 g/L FeCl_3_.

The R_lab_ in *C. sorokiniana* with AlCl_3_ was 91.1 ± 0.5% at the dose of 0.1 g/L and 90.4 ± 0.4% at the dose of 0.2 g/L (Figure 1g). For the lowest tested dose that was used (0.05 g/L), R_lab_ was only 51.7 ± 3.1%. The same tendency was observed for FeCl_3_, where higher doses of 0.4 and 0.2 g/L resulted in R_lab_ values of 94.7 ± 0.1% and 91.1 ± 0.5%, respectively, and the minimum dose, 0.1 g/L of AlCl_3_, resulted in an R_lab_ of 87.1 ± 0.5%. All tested doses of FeCl_3_ reached the plateau after 30 min of treatment (Figure 1h). Furthermore, biomass treated with highest dose of FeCl_3_ (0.4 g/L) noticeably turned yellow, which was not the case with the lower doses (see Figure A2, Appendix B). Unlike AlCl_3_ and FeCl_3_, data of the R_lab_ using chitosan showed that at the minimum dose tested (0.06 g/L), the R_lab_ was higher (52.6 ± 0.5%) compared with the very low R_lab_ values obtained at higher doses: R_lab_ = 7.2 ± 0.7% at 0.08 g/L and 8.0 ± 0.4% at 0.1 g/L of chitosan (Figure 1i).

### 3.2. Evaluation of Flocculants in Pilot Scale

Based on the results in the laboratory-scale flocculation, the most effective doses of three different flocculant were tested in an outdoor cultivation system. Cultures were scaled up from a 250 L to a 1600 L raceway and maintained semi-continuously to obtain a sufficient culture volume to test three different doses of the three different flocculants with each strain. Before each flocculation experiment a culture volume of 400 L was evenly distributed to four 100 L vertical column photobioreactors (PBRs), and the selected doses of the flocculants were dissolved as described in the Section 2. Mixing was applied for 20 min, and then the culture was left to settle for 180 min. In the last, (fourth) experiment, the best-performing doses of each flocculant were once more tested to confirm the obtained results. For every strain, four pilot-scale experiments were performed, in three columns each.

#### 3.2.1. Effect of Flocculants in *T. striata* at Pilot Scale

**Figure 2:**
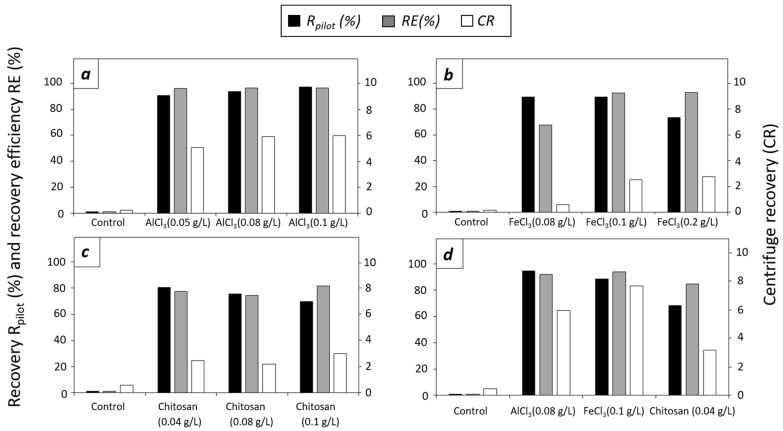
Data of *T. striata* recovery (R_pilot_, %, black columns), recovery efficiency (RE, %, gray columns), and centrifuge recovery (CR, white columns). (**a**) Control and doses of 0.05, 0.08, and 0.1 g/L of AlCl_3_. (**b**) Control and doses of 0.08, 0.1, and 0.2 g/L of FeCl_3_. (**c**) Control and doses of 0.04, 0.08, and 0.1 g/L of chitosan. (**d**) Control and best performing doses of AlCl_3_ (0.08 g/L), FeCl_3_ (0.1 g/L), and chitosan (0.04 g/L).

The results of the pilot-scale flocculation experiments in *T. striata* cultures are shown in Figure 2.

In the first pilot-scale flocculation experiment, AlCl_3_ was tested at doses of 0.05, 0.08, and 0.1 g/L (Figure 2a)**.** For all three different doses, an interphase between the supernatant and the flocs was formed, and the pilot-scale recovery (R_pilot_, %) was higher than 85%. Floc formation increased the centrifuge recovery of the biomass, which was 5.10, 5.95, and 6.02 for AlCl_3_ at 0.05, 0.08, and 0.1 g/L, respectively, compared with the centrifuge recovery of 0.20 for the untreated culture (control). Also, the recovery efficiency was RE ≥ 96%. In the second experiment, FeCl_3_ was tested at doses of 0.08, 0.1, and 0.2 g/L (Figure 2b)**.** Similar to AlCl_3_, the three doses of FeCl_3_ formed an interphase between the supernatant and the flocs, but the same doses of 0.08 and 0.1 g/L FeCl_3_ resulted in lower R_pilot_ values of 85.9 and 89.3%, respectively, when compared with AlCl_3_. Due to the effect of the flocculant, the centrifuge recovery was improved from 0.19 for the control culture to 0.59, 2.58, and 2.78 for the 0.08, 0.1, and 0.2 g/L FeCl_3_, respectively. The higher doses resulted as well in recovery efficiencies higher than 92%, and the minimum dose resulted in a recovery of 67.6%. In the third experiment, doses of 0.04, 0.08, and 0.1 g/L of chitosan were tested (Figure 2c). Different from the tests performed with the chemical flocculants, the flotation of some flocs was observed. The R_pilot_ of all different doses was lower than 81%. The maximum R_pilot_ = 80.5% was obtained at 0.04 g/L of chitosan. As observed previously with AlCl_3_ and FeCl_3_, the centrifuge recovery improved from 0.56 for the control culture to the maximum value of 3.09 for 0.1 g/L chitosan. Recovery efficiencies were higher than 74% for all tested doses of chitosan. The harvested paste was visually different from other cases and had a viscous consistency and appearance compared with the paste obtained with AlCl_3_ and FeCl_3_. The best doses, i.e., 0.08 g/L for AlCl_3_, 0.1g/L for FeCl_3_, and 0.04 g/L for chitosan, were repeated in the fourth experiment (Figure 2d). The R_pilot_ was 94.6% for AlCl_3_, 88.4% for FeCl_3_, and 68.3% for chitosan. Centrifuge recovery was 5.96 for AlCl_3_, 7.67 for FeCl_3_, and 3.18 for chitosan. A recovery efficiency higher than 91% was obtained for AlCl_3_ and FeCl_3_ and 84.8% for chitosan. For the four experiments the same concentration factor of 14.29 was obtained due to the same harvested volume of 7.0 L.

#### 3.2.2. Effect of Flocculants in *D. tertiolecta* at Pilot Scale

**Figure 3:**
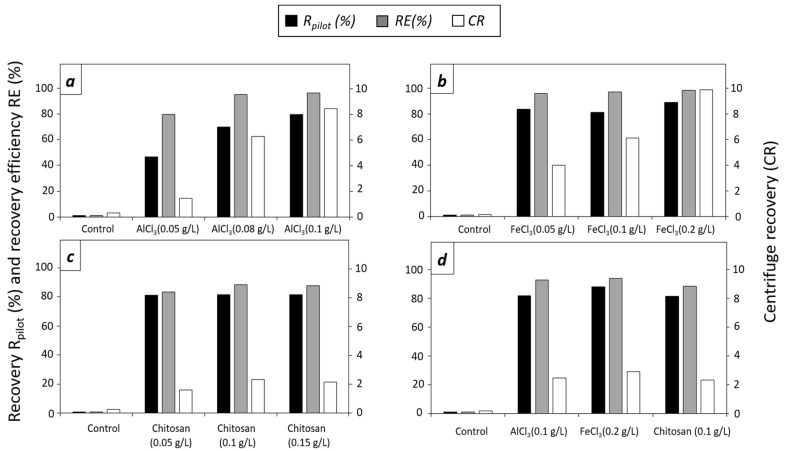
Data of *D. tertiolecta* pilot-scale recovery (R_pilot_, %; black columns), recovery efficiency (RE, %; gray columns), and centrifuge recovery (CR, white columns). (**a**) Control and doses of 0.05, 0.08, and 0.1 g/L of AlCl_3_. (**b**) Control and doses of 0.05, 0.1, and 0.2 g/L of FeCl_3_. (**c**) Control and doses of 0.05, 0.01, and 0.15 g/L of chitosan. (**d**) Control and best doses of AlCl_3_ (0.1 g/L), FeCl_3_ (0.2 g/L), and chitosan (0.1 g/L).

The results of the flocculation experiments with *D. tertiolecta* at a pilot scale are shown in Figure 3.

**Figure A4:**
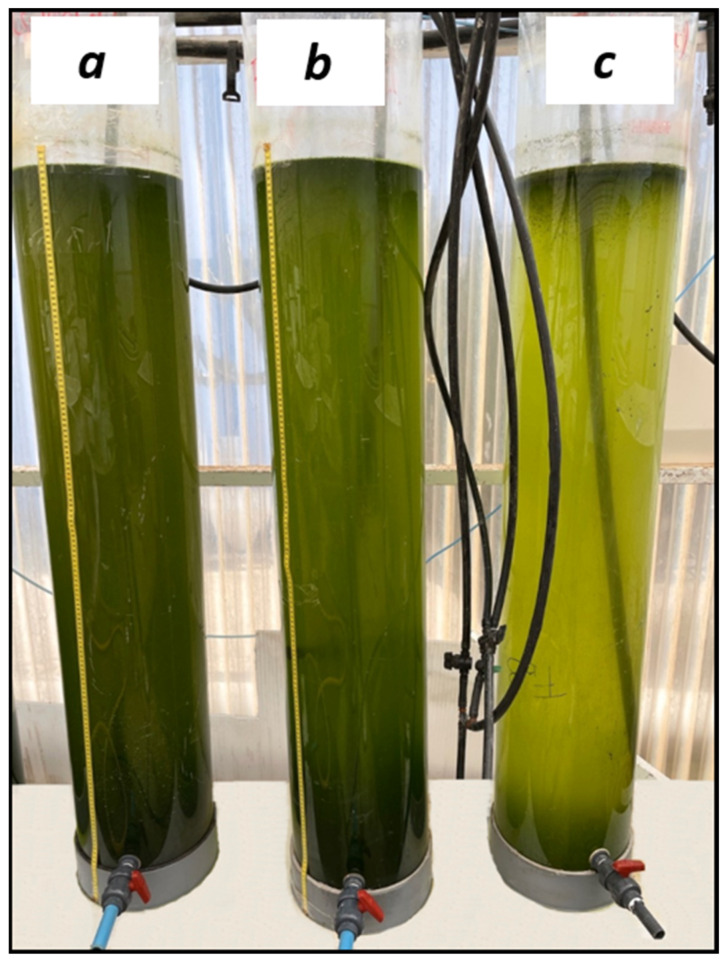
Floc formation and flotation with different concentrations of FeCl_3_ at pilot-scale flocculation of *D. tertiolecta*: (**a**) 0.05 g/L FeCl_3_, (**b**) 0.1 g/L FeCl_3_, and (**c**) 0.2 g/L FeCl_3_.

AlCl_3_ doses tested with *D. tertiolecta* cultures were 0.05, 0.08, and 0.1 g/L, with R_pilot_ values of 46.7%, 70.0%, and 79.8%, respectively (Figure 3a). The addition of AlC_3_ enhanced the harvesting recovery, with 0.30 for the control culture, with the maximum value of 1.45 8.47 for 0.1 g/L of AlCl_3_. The recovery efficiency of the highest doses of AlCl_3_ (0.08 g/L and 0.1 g/L) were higher than 95%, and the recovery efficiency was 79.7% for the lowest dose (0.05 g/L). For AlCl_3_ and FeCl_3_ at higher doses a clear interphase was formed after 180 min. When AlCl_3_ was applied, the flocs completely settled down at the bottom of the PBR. Differently, some flocs floated when FeCl_3_ was applied at the highest dose (0.2 g/L) (see Figure A4, Appendix B). The clear interphase formed, and only 7.0 L of the culture was harvested, including the floating flocs, determining a concentration factor of 14.29 for all the doses of AlCl_3_ and FeCl_3_. Figure 3b shows the different doses of FeCl_3_ tested with *D. tertiolecta*. The obtained R_pilot_ values were from 83.6% to 89.1% for an increasing FeCl_3_ concentration of 0.05 g/L to 0.2 g/L, respectively. Centrifuge recovery was higher with maximum doses (9.97 for 0.2 g/L FeCl_3_) compared with CR = 0.16 obtained with the control culture, similar to what was observed for AlCl_3_. The obtained recovery efficiencies were higher than 95.0% for all tested doses. Concerning chitosan, doses of 0.05, 0.1, and 0.15 g/L were tested (Figure 3c). In contrast to AlCl_3_ and FeCl_3_, the concentration factor for chitosan was lower due to the lack of formation of the clear interphase between flocs and the supernatant and because of the partial flotation of the biomass. For that reason, the amounts of harvested cultures were 17 L (10 L more than in the experiments with the chemical flocculants). R_pilot_ was lower than 82% but changed insignificantly with increasing chitosan doses (R_pilot_ = 81.1, 81.6, and 81.4% for 0.05, 0.1, and 0.15 g/L of chitosan). Centrifuge recoveries were obtained for all three doses tested due to the solid consistency of the biomass, with the maximum value of 2.33 for 0.1 g/L. As for *T. striata*, flocculation of *D. tertiolecta* was repeated, applying the most effective doses of the flocculants: 0.1 g/L for AlCl_3_, 0.2 g/L for FeCl_3_, and 0.1 g/L for chitosan (Figure 3d). The R_pilot_ was 81.7% for 0.1 g/L AlCl_3_ and 87.9% for 0.2 g/L FeCl_3_. The obtained centrifuge recovery was 2.50 for 0.1 g/L AlCl_3_ and 2.92 for 0.2 g/L FeCl_3_. A recovery efficiency higher than 92% was obtained for AlCl_3_ and FeCl_3_.

#### 3.2.3. Effect of Flocculants in *C. sorokiniana* at Pilot Scale

**Figure 4:**
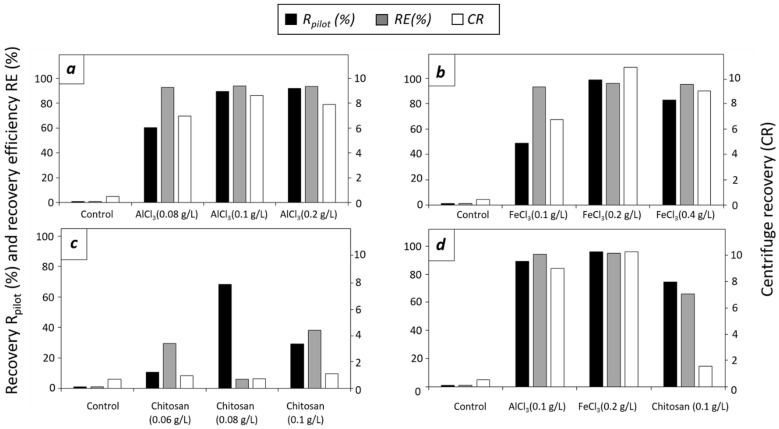
*C. sorokiniana* recovery (R_pilot_, %; black columns), recovery efficiency (RE, %; gray columns), and centrifuge recovery (CR, white columns). (**a**) Control and doses of 0.08, 0.1, and 0.2 g/L of AlCl_3_. (**b**) Control and doses of 0.1, 0.2, and 0.4 g/L of FeCl_3_. (**c**) Control and doses of 0.06, 0.08, and 0.1 g/L of chitosan. (**d**) Control and best doses of AlCl_3_ (0.1 g/L), FeCl_3_ (0.2 g/L), and chitosan (0.1 g/L).

The results of the pilot-scale flocculation experiments on *C. sorokiniana* cultures are shown in Figure 4.

**Figure A5:**
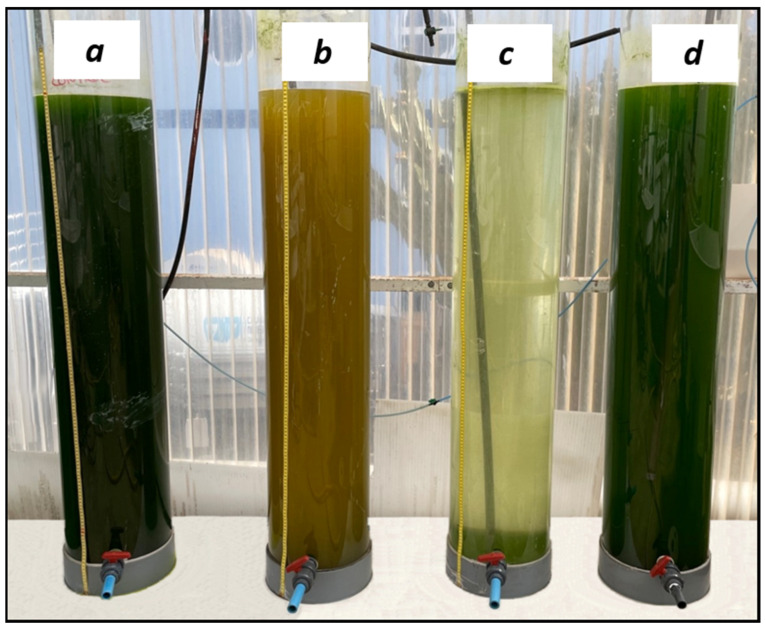
Color changes of the supernatant with different concentrations of FeCl_3_ at pilot-scale flocculation of *C. sorokiniana*: (**a**) Control culture without flocculants, (**b**) 0.4 g/L FeCl_3_, (**c**) 0.2 g/L FeCl_3_, and (**d**) 0.1 g/L FeCl_3_.

Three different doses (0.08, 0.1, and 0.2 g/L) of AlC_3_ were tested (Figure 4a). All three doses formed an interphase between the flocs and the supernatant with R_pilot_ higher than 85% for AlCl_3_ doses of 0.1 and 0.2 g/L. The centrifuge recovery of the control culture of *C. sorokiniana* was 0.53, while the highest centrifuge recovery of 8.68 was obtained for 0.1 g/L of AlCl_3_. For the different doses of AlCl_3_, the RE was higher than 92%. In the experiments using FeCl_3_, doses of 0.1, 0.2, and 0.4 g/L were tested (Figure 4b). The obtained R_pilot_ values were higher than 85% only for 0.2 g/L of FeCl_3_. Yellow coloration of the culture medium was observed with 0.4 g/L FeCl_3_ (see Figure A5, Appendix B). Centrifuge recovery was 6.79, 10.95, and 9.09 for doses of 0.1, 0.2, and 0.4 g/L of FeCl_3_, respectively, while RE values were higher than 93%. The concentration factor was 14.29 for AlCl_3_ and FeCl_3_ as a consequence of the centrifugation of 7.0 L of flocs. Figure 4c shows flocculation data with chitosan. The R_pilot_ was lower than 69% using all three doses. Centrifuge recoveries were low for all three doses (0.96 for of 0.06 g/L, 0.72 for 0.08 g/L, and 1.10 for 0.1 g/L). RE values were also lower compared with recovery efficiencies obtained with chemical flocculants, with a maximal value of 38% for 0.1 g/L of chitosan. Due to the lack of a clear visible observed interphase, 35 L of flocs were harvested, resulting in the concentration factor of 2.86. When chitosan was applied, the flotation of some flocs was observed. For the last experiment, the best-performing doses of AlCl_3_ (0.1 g/L), FeCl_3_ (0.2 g/L), and chitosan (0.1 g/L) were tested (Figure 4d). Centrifuge recoveries for the best doses were 0.53 for the control culture, 9.0 for 0.1 g/L of AlCl_3_, 10.29 for 0.2 g/L of FeCl_3_, and 1.55 for 0.1 g/L of chitosan. In terms of R_pilot_ and RE, values higher than 89% were obtained for AlCl_3_ and FeCl_3_, and a value of 65.8% was obtained for chitosan. The concentration factor for chitosan was 7.14 because 14 L was harvested, compared with 35 L in the previous experiments.

### 3.3. Quality of the Flocculated Biomass

The Al, Fe, ash, and water contents determined on the fresh microalgal paste harvested from the three microalgae tested at the pilot scale with the most effective dose of each flocculant for *T. striata*, *D. tertiolecta*, and *C. sorokiniana* are shown in Table 1. This table shows that Al and Fe significantly accumulated in the harvested biomass treated with the chemical flocculants AlCl_3_ and FeCl_3_ for all three selected microalgae. The highest concentrations of Al and Fe in the corresponding flocculation treatment (40,429.3 ± 585.7 and 59,462.0 ± 2339.3 ppm) were observed in *C. sorokiniana*, while the lowest concentrations (34,335.7 ± 5041.1 and 53,237.0 ± 4847.4 ppm) were detected in the *D. salina* biomass. Accordingly, the lowest concentration magnification values in the biomass (i.e., the ratio between the concentration of the metal in the respective treatment and the concentration in the control) were found in *T. striata* (28 and 33 for Al and Fe, respectively), whereas the highest magnification values were detected in *C. sorokiniana* for Al (288) and in *D. tertiolecta* for Fe (117). The Fe content in the FeCl_3_-treated biomass was significantly higher than the Al content in the AlCl_3_-treated biomass for all three microalgae, as expected by the higher doses used for flocculation (see Figure 2d, Figure 3d and Figure 4d). Ash content on a dry weight basis (range: 7.2–35.4%) significantly correlated with flocculant dose (r = 0.75) and Fe content in the biomass (r = 0.75) and was significantly higher in treatments with the chemical flocculants compared with the control for all three microalgae. On the other hand, the ash content in chitosan-treated biomass did not differ significantly from the control for all the tested strains (Table 1). Interestingly, the water content (range of 70.3–89.2%) also positively correlated with the flocculant dose (r = 0.73) and was significantly higher in all the biomass treated with flocculants compared with the control for all the microalgae (Table 1). The water content (range of 70.3–89.2%) also positively correlated with the flocculant dose (r = 0.73) and was significantly higher in all the biomass treated with flocculant compared with the control for all tested species. The water content in the flocculant-treated biomass varied significantly among the three algal species, with the lowest (80.0 ± 1.4%) and the highest (87.4 ± 1.5%) values obtained for *T. striata* and *C. sorokiniana*, respectively.

A detailed elemental analysis of biomass is presented in Appendix A (Table A1, Table A2 and Table A3) including minerals and heavy metals. No traces of Hg, Co, As, and Se were detected in any of the tested samples.

### 3.4. Economic Viability Assessment of Down-Streaming in One-Step and Two-Step Process

To compare the cost of microalgae harvesting by traditional centrifugation and two steps (using flocculants followed by the centrifugation of the concentrate), we calculated the cost of each flocculant at the best-performing dose for each microalga (Table 2).

For the chemical flocculants (AlCl_3_ and FeCl_3_), the harvesting cost declined compared with the traditional single-step centrifugation process. For *T. striata*, the price reductions were 89.7% and 86.3% using AlCl_3_ and FeCl_3_, respectively. For *D. tertiolecta* and *C. sorokiniana*, the reduction was 88.0% by using AlCl_3_ and 79.5% by using FeCl_3_. In the case of chitosan, the price was higher for all microalgae and was also higher in the flocculation of *D. tertiolecta* and *C. sorokiniana* compared with *T. striata* due to the lower values of the concentration factors, 5.88 and 7.14, respectively.

## 4. Discussion

Microalgae are technologically promising microorganisms with great biotechnological potential and ecological benefits, used as sustainable feedstock for biofuel production and diverse valuable bioresources with commercial interest [50,51,52]. The high cost of harvesting (up to 30% of the total production costs [53]) has urged the necessity to find new economically sustainable harvesting techniques.

When microalgae are removed from the photobioreactor and placed into a tank, the absence of mixing, pumping, and air bubbling combined with natural gravity can lead to spontaneous biomass settling known as autoflocculation [10]. Autoflocculation is a simple and chemical-free process, but it is time consuming, unreliable, and suitable only for a few autoflocculating species [54]. The autoflocculating affinities of the three microalgae under investigation are presented in Figure A1 (Appendix A). The R_lab_ obtained for *T. striata* was 47.6%, suggesting that this species can be considered as an auto-settling microalga, which was already reported for *Tetraselmis suecica*, which readily autoflocculates with recovery efficiencies of 40–60% [51]. Moreover, the autoflocculating nature of *Tetraselmis* sp. was used to easily pre-concentrate large-scale cultures by leaving them to settle in a 1.0 m^3^ sedimentation tank [11]. Although gravity sedimentation is a low-cost harvesting method, it presents some downsides such as the long operational time, which can cause deterioration of the biomass (e.g., because of the significant increase in the bacterial load, which can make biomass unsuitable for some applications), and the impossibility to apply it in low-density cultures [52,55]. The R_lab_ of *C. sorokiniana* was 8.2 ± 1.46%, which was in accordance with other studies [29,44] that recognize *Chlorella* sp. as non-settling microalgae. For *D. tertiolecta* the R_lab_ was 28.6 ± 8.56%, which was in between the values for *T. striata* and *C. sorokiniana*, suggesting this was a mildly auto-settling species. This result was different from the 5% flocculation of *D. tertiolecta* reported by [56] and was most probably due to the high pH of the culture in the referenced work (pH = 10.7) compared with the pH reported in the present study (7.4 ± 0.28) and also due to the different culture medium, which can affect flocculation [57].

Free polyvalent metal cations such as Al^3+^, Fe^3+^, Mg^2+^, and Zn^2+^ and their various positively charged hydrolysates can neutralize the negative charges on the surface of algal cells and promote the collision and aggregation of cells and floc formation [14]. The results of the flocculation in the laboratory-scale experiments are presented in Figure 1. We can conclude that AlCl_3_ was more effective than FeCl_3_, in all tested microalgae species except *T. striata*. Aluminum salts have lower molecular weight, higher solubility [15], and higher charge density [17] than ferric salts, which results in a lower flocculation efficiency of Fe salts [30]. These Al^3+^ characteristics result in the extended molecular conformation that promotes bridging between cells, thus improving the charge neutralization of the microalgae cell surfaces [58]. Also, the R_lab_ of some of the highest AlCl_3_ doses applied (0.20 g/L for *T. striata* and *C. sorokiniana*) was lower compared with the R_lab_ obtained at 0.1 g/L AlCl_3_ due to the saturation phenomenon, which may occur at high doses of flocculant because of the strong repulsion between polyvalent cations and monolayer adsorption on the cell surface leading to re-stabilization of the cell suspension [59].

Several studies have tested different concentrations of aluminum and ferric salts as flocculants in microalgae cultures, at the lab scale. In the present work, the maximum R_lab_ values obtained with *T. striata* were 96.9 ± 0.19% and 96.7 ± 0.46% using 0.1 g/L of AlCl_3_ and 0.08 g/L of FeCl_3_, respectively. These results were similar to the data reported for *Tetraselmis* sp. with recoveries of 85.6% using 1.2 g/L of Al_2_(SO_4_)_3_ (at pH = 5.0–6.0) and 92.6% using 0.7 g/L of Fe_2_(SO_4_)_3_ (at pH = 4.0–8.0) after 30 min of flocculation combined with air flotation [60]. Flocculation of *Tetraselmis tetrahele* using 0.2 g/L of Al_2_(SO_4_)_3_ resulted in flocculation efficiencies of 98.65% [61]. In this study, the maximum R_lab_ values obtained for *D. tertiolecta* were 95.6 ± 0.16% and 97.8 ± 1.1% with 0.1 g/L AlCl_3_ and 0.2 g/L of FeCl_3_, respectively, which was in accordance with the reported 93% recovery of *D. tertiolecta* using 0.021 g/L of FeCl_3_ and the almost 100% recovery with 1.26 mg/L of AlCl_3_ and Al(SO_4_)_3_·18H_2_O [56]. A recovery of 85% was obtained with *D. salina* cultures using 0.13 g/L of FeCl_3_ [55].

For *C. sorokiniana* the maximum R_lab_ values were 90.4 ± 0.4% and 94.8 ± 0.1% using 0.2 g/L AlCl_3_ and 0.4 g/L of FeCl_3_, respectively, which was similar to the 98% recovery reported for *C. sorokiniana* flocculated with 0.25 g/L of Fe_2_(SO_4_)_3_ [55]. Lower flocculation efficiency (60–70%) was reported for *C. vulgaris* flocculated with 0.4 and 0.5 g/L FeCl_3_ at the laboratory scale and pH = 7 [62]. Three different flocculants were tested on *Chlorella vulgaris*: culture broth from *Paenibacillus* sp., aluminum sulfate (2 mg/L), and polyacrylamide (2 mg/L) with biomass recoveries of 83%, 72%, and 78%, respectively [28]. Lower biomass recovery using aluminum salt in this study compared with our results can be attributed to the much lower Al concentration that the authors used (2 mg/L vs. 0.1 g/L in this study). It was also reported for *C. minutissima* that 0.5 g/L of AlCl_3_ and FeCl_3_ were the most efficient doses at the laboratory scale [30]. Another study reported that 15-day-old *Chlorella vulgaris* was flocculated efficiently (with 80% recovery) when mixed with a *Scotelliopsis reticulata*, which improved its sedimentation efficiency by 52% [27]. The use of biological flocculants derived from other microalgal species is a good alternative to chemical flocculation, although it has limited applications—especially for *Chlorella vulgaris* used for food products as another species might not be permitted for human nutrition. It was reported that adding 0.79 mM FeCl_3_ to *Botryococcus braunii* and using a mixing time of 180 s achieved a biomass recovery of 90.6 % [37]. Aluminum sulfate and ferric chloride at concentrations of 70 μM added to *Nannochloropsis* sp. resulted in recovery efficiencies of 80% and 90%, respectively, at pH = 6.5 to 8.0 [34].

Chitosan is a natural polymer with a high cationic charge density [20]. The mechanism involved in chitosan flocculation is the neutralization of the negative charge of the microalgal cells, which induces flocculation by adsorption bridging of the polymer chain [14]. Chitosan contains amino groups that at lower pH have a high positive charge and attach to the microalgae, which have negatively charged cell surfaces, and can be used as a viable flocculant for both the *Chlorella* and *Scenedesmus* species, as well as for the wastewater treatment [63]. The mode of action of chitosan includes adsorption, bridging, sweeping, and charge neutralization, and it is more effective in acidic conditions due to its linear arrangement surrounded with positively charged deacetylated groups, resulting in effective charge neutralization of microalgae [64]. The effectiveness of chitosan generally decreases in the seawater medium because of its high ionic strengths, which can screen positive charges of chitosan, thus partially preventing the polymer from interacting with the algal cells [65].

In this study the maximum R_lab_ obtained with *T. striata* was 73.2 ± 1.07% with 0.08 g/L of chitosan. It was reported that the addition of 4.0 and 5.0 g/L of chitosan to *Tetraselmis* sp. combined with dissolved air flotation resulted in more than 80% efficiency [60]. In *D. tertiolecta* flocculation experiments with 0.15 g/L chitosan the recovery R_lab_ = 74.0 ± 1.2% was higher than R_lab_ = 42.2 ± 0.7% for 0.2 g/L chitosan. This phenomenon was already reported by Beach et al. (2012), where 0.1 g/L chitosan flocculated *N. oleoabundans* with the 95% biomass recovery, while higher doses of chitosan were not beneficial and increased the turbidity of the *N. oleoabundans* cultures [33]. A similar trend of the decrease in flocculation efficiency when increasing the dose of chitosan was also observed in *D. salina* cultures [66]. *Chlorella sorokiniana* was successfully flocculated using 10 mg/g_dw_ of chitosan with an efficiency of 99% at a pH = 6 [25]. Chitosan at a concentration of 0.25 g/L achieved more than 90% biomass recovery of *Chlorella vulgaris* in 10 min [67]. The authors further used spent medium after chitosan flocculation for the recultivation of microalgae, which demonstrated robust growth in comparison with the cultures recultivated in other recycled medium [67]. Chitosan was used to flocculate marine microalgae *Nannochloropsis* sp. and *N. oculata* [68,69]. Chitosan at 125 μM and pH = 8.5 achieved 37.7% biomass recovery in *Nannochloropsis* sp. culture [34]. On the other hand, recovery using chitosan was 90% in *Phaeodactylum tricornutum* cultures, which indicated that flocculation with chitosan might be strongly species dependent [32,34]

In the three tested microalgae, the doses of chitosan applied at the laboratory scale with the highest recoveries were the intermediate dose in the case of flocculation with *T. striata* (0.08 g/L), the maximum dose applied to *D. tertiolecta* (0.15 g/L), and the minimum dose with *C. sorokiniana* (0.06 g/L). At the higher concentrations of chitosan, cells tended to strongly repel each other because of the large positive charge of the chitosan-adsorbed particles [20]. In addition to the distinct conditions between outdoor and indoor experiments, the differences between recoveries, in this study, could be attributed to the mixing time. While in laboratory tests the cultures with the addition of chitosan were mixed for 2 min using a vortex mixer, in the outdoor PBRs the cultures treated with chitosan were mixed for 20 min using gas bubbling, which was in accordance with [20], which reported the optimal removal of *Chlorella* sp. with chitosan using a mixing time of 20 min. Also, the mixing speed can affect flocculation as rapid mixing can induce destabilization of the formed colloids and hinder particle aggregation [70]. In addition, when chitosan was applied in our pilot-scale experiments, partial flotation of the flocs was observed, as was already described in [20]. Further treatment of the flocs is necessary. This additional process will inevitably lead to an increase in potential costs.

Currently, only a few studies are available about the use of flocculants in the pilot-scale microalgae cultures [23,31,35]. In our study the biomass recovery values (R_pilot_, %) of 100 L cultures were as follows: (1) for *T. striata*, R_pilot_ = 94.6% for 0.08 g/L AlCl_3_, 88.4% for 0.1 g/L FeCl_3_, and 68.3% for 0.04 g/L chitosan; (2) for *D. tertiolecta*, R_pilot_ = 81.7% for 0.1 g/L AlCl_3_, 87.9% for 0.2 g/L FeCl_3_, and 81.6% for 0.1 g/L chitosan; and (3) for *C. sorokiniana*, R_pilot_ = 89.6% for 0.1 g/L AlCl_3_, 98.6% for 0.2 g/L FeCl_3_, and 68.3% for 0.1 g/L chitosan. These recoveries were similar to those reported for 200 mg/L FeCl_3_ and 250 mg/L AlCl_3_ in 2 L *Scenedesmus obliquus* cultures with efficiencies of 80.2% and 95%, respectively [35]. The authors further scaled up the cultures and reported 75% and 60% flocculation efficiency for the pilot-scale (1000 L) cultures of *S. obliquus* and *C. vulgaris*, at pH = 12 [35]. Pilot-scale flocculation using NaOH (to 8 mM) and polyelectrolyte (non-ionic polymer Magnafloc LT-25 at 0.5 mg/L) was performed in *T. pseudonana* 130 L cultures with a biomass recovery of 89% and were concentrated to 5 L volume in 15 min time with a concentration factor of 26 [23], which was higher than the concentration factor of 14.29 for 0.1 g/L of AlCl_3_ and FeCl_3_, reported in this study. This can be explained by the higher sweeping power achieved through the combined effect of the high pH and the industrial polyelectrolyte. The authors also tested various microalgae species at larger volumes (10–1000 L) including *Tetraselmis suecica*, *C. calcitrans*, *C. muelleri*, *Skeletonema* sp., *R. salina*, *A. septentrionalis*, *Nitzschia closterium*, and *C. muelleri* and concluded that all these species were successfully flocculated with similar efficiencies (≥85%) as observed for *T. pseudonana* with the combined effect of 8 mM NaOH and 0.5 mg/L of non-ionic polymer. Pilot-scale flocculation of *Dunaliella salina* with 80% recovery was achieved by the addition of 0.02 M NaOH, which resulted in the precipitation of Mg(OH)_2_ and the seeping of algal cells from the seawater medium [31]. This method required the presence of sufficiently high Mg^2+^ concentration in the medium (conc. [Mg^2+^] > 0.1 mM) [71]. Pilot-scale flocculation using 35 mg/g_dw_ of industrial cationic polymer on 350 L cultures of *Chlorella vulgaris* demonstrated that flocculation efficiency increased from 35% in the early exponential phase to 80% in the late stationary phase [26]. This was explained by the zeta potential change from the more negative to less negative (closer to zero) with culture aging, which implied faster flocculation in mature cultures as there was less need for the cationic polymer to achieve charge neutralization [26]. Flocculation of *Desmodesmus brasiliensis* by microbial bio-flocculant resulted in a flocculation efficiency higher than 98% [72]. Further study of cyanobacterium *Synechocystis* sp., the freshwater *Chlorella vulgaris*, and the marine *Phaeodactylum tricornutum* at the laboratory and pilot scales (350 L) showed that chitosan could not effectively flocculate *C. vulgaris* during the exponential phase but reached a maximum of 74% recovery with 0.06 g/L chitosan during the stationary phase [16], which was similar to the chitosan flocculation recoveries of the 100 L cultures in this study (68.3–81.6% recovery using 0.04–0.1 g/L of chitosan).

Centrifugation is a common, fast, and effective method to harvest the majority of microalgal strains [10,15,52,73]. For some strains, centrifugation is sufficient as a one-step solid–liquid separation process, while other strains require a pre-concentration step [74]. Nevertheless, centrifugation has some downsides, including the high cost of equipment and maintenance, the high energy demand, and the risk of cell damage, which make this method unsuitable for its application to large-scale cultivation systems [10,17,52,75]. Centrifuges are normally set to maximize harvesting efficiency; however, cost-effective microalgal harvesting may not match this target [76]. To achieve high harvesting efficiencies, longer retention times in the bowl are needed to enable microalgae sedimentation due to the small size of the cells [15]. While 100 L were harvested from the untreated culture to recover the whole biomass, in this study, the volume of cultures treated with the different flocculants sharply decreased due to the improved concentration factor (14.29 for AlCl_3_ and FeCl_3_ and 7.14 and 5.88 for chitosan). As the harvested volume was reduced by 93% for AlCl_3_ and FeCl_3_ and 86% for chitosan, the energy consumption of the centrifugation decreased. This was in accordance with the reported 20- to 50-fold decrease, or 95–98% reduction, of the volume to be processed after the flocculation of *C. sorokiniana* with chitosan [25]. The use of flocculants before centrifugation in this study reduced the harvesting cost by 89.74% and 86.33% for *T. striata* using AlCl_3_ and FeCl_3_, respectively, while the reduction was 88.03% when using AlCl_3_ and 79.49% using FeCl_3_ for *D. tertiolecta* and *C. sorokiniana*. Although the use of chitosan generally increased the harvesting cost for all three tested microalgal species, the harvesting cost for *T. striata* using this natural flocculant was 3.15 EUR/m^3^, which was lower compared with 7.88 and 7.84 EUR/m^3^ calculated for *D. tertiolecta* and *C. sorokiniana*, respectively. This result suggested that chitosan could be a viable flocculating agent for auto-settling microalgal strains due to its biodegradability and biocompatibility [77]. It was recently reported that the chitosan cost for flocculation of 1000 kg of *Chlorella vulgaris* biomass was USD 21–35 [78]. The authors predicted the optimum parameters using a response-surface model (pH = 5, flocculation time of 45 min, 10 mg/L chitosan, and recovery efficiency 99.10%), which could somewhat underestimate the final costs [78]. The costs per 1000 kg of *C. vulgaris* and *S. obliquus* biomass obtained using 3–5 mg/L of chitosan pre-concentration were USD 176.81 and USD 106.95, respectively [54]. In comparison, it was reported that pH-induced flocculation of *C. vulgaris* and *S. obliquus* at a large scale cost USD 500–530 per 1000 kg of biomass [35]. Although the use of chitosan, in our case, increased the harvesting costs compared with the chemical flocculants, centrifuge recovery increased for all the microalgae. Indeed, the high centrifuge recovery (6.6 times higher than the control) in the case of *T. striata* can partially compensate for this cost increase.

Although our study demonstrated that flocculation with AlCl_3_ and FeCl_3_ reduced the harvesting cost for three different microalgae, the high residual aluminum and iron content in the AlCl_3_- and FeCl_3_-treated biomass (up to 40,000 and 59,000 ppm in *C. sorokiniana*, respectively; see Table 1) hamper its use for human consumption and animal feeding. The European Food and Safety Authority advises a tolerable weekly intake of 11 mg/kg of body weight per week for Al [79] and a tolerable upper intake level of 45 mg/day (1300 ppm) for Fe [80], while the limit of Fe for generic animal consumption is 750 mg/kg of the complete feed content [81]. Al and Fe in this study resulted to be the highest for *C. sorokiniana* and the lowest for *D. tertiolecta* biomass, when equal doses of flocculant were applied, indicating that specific characteristics of the flocculated strain (e.g., the surface charge and cell size [57]) could enhance metal accumulation in the biomass. The highest metal content that we found in *C. sorokiniana* was also confirmed by several studies that reported the high biosorption capacity of this microalgal species [82,83]. Moreover, the use of chemical flocculants led to a significant dose-dependent increase in the ash content of the biomass, not only due to the accumulation of the Al and Fe added at the beginning of the flocculation process but also due to the accumulation of the most abundant elements present in the culture medium due to the co-precipitation induced by the added flocculants salts [45]. On the other hand, the metal and ash contents in the chitosan-treated biomass were comparable to the control for all three tested strains. This result was confirmed by the previous study that reported the low ash content in the commercial chitosan [84], which could be used in the pre-concentration step for the production of high-valuable products. Although the EU Commission does not directly limit the content of Fe and Al and regulates only the content of arsenic, cadmium, and lead in animal feed [85], an economically viable solution to remove the excess metals has already been reported [14]. Contamination of the biomass by metals could be resolved by adjusting the pH of the culture due to the chemical properties of aluminum- and iron-based flocculants. Indeed, iron-based flocculants dissolved in water tend to form precipitates of metal hydroxide. These iron-based precipitates will release Fe ions and be redissolved by decreasing the pH, which removes the iron precipitates from the biomass and also recovers the Fe ions. Aluminum-based flocculants could also be recovered using this methodology due to their similar chemical properties [14]. It has already been reported that reducing the pH with sulfuric acid in the culture of *Chlorella* sp. flocculated with Fe_2_(SO_4_)_3_ allows the release of the sediment attached to the microalgae, resulting in metal-free biomass. Additionally, the remaining acidic solution containing the recovered iron ions can be reused three more times for the flocculation with an efficiency of up to 98% [86]. Furthermore, the addition of 0.1 M HCl after harvesting *Scenedesmus acuminatus* flocculated by Al_2_(SO_4_)_3_·18H_2_O reduced the Al content approximately six times (from 59.74 mg/g to 0.11 mg/g) [86]. In addition, the recovered Al ions were concentrated 25 times and reused 5 more times for harvesting *S. acuminatus*, with harvesting efficiencies higher than with the fresh flocculant possibly due to the presence of extracellular polymeric substances in the recovered coagulant solution. The cost of the chemical flocculant decreased by 50% after five times recycling [14,87]. Redissolving the ions by decreasing the pH is a promising technique to remove the excess metals from the biomass, which merits further investigation.

## 5. Conclusions

Based on the obtained results, we can conclude that the flocculation step prior to centrifugation can be a viable solution for large-scale microalgae culture harvesting. The addition of flocculants to the 100 L cultures of three selected strains resulted in biomass recoveries of 94.6%, 81.7%, and 89.6% for AlCl_3_ in *T. striata*, *D. tertiolecta*, and *C. sorokiniana*, respectively, with similar recoveries for FeCl_3_. The biomass recoveries using chitosan were slightly lower in all tested species. The use of flocculants before centrifugation reduced the harvested volume by 93% for AlCl_3_ and FeCl_3_ and 86% for chitosan and consequently reduced the harvesting cost by 89.74% and 86.33% for *T. striata* using AlCl_3_ and FeCl_3_, respectively. The cost reduction was 88.03% using AlCl_3_ and 79.49% using FeCl_3_ for *D. tertiolecta* and *C. sorokiniana*. The use of chitosan generally increased the harvesting cost for all three tested microalgal species. Nevertheless, the harvesting cost for *T. striata* using chitosan was 3.15 EUR/m^3^, which was much lower compared with 7.88 and 7.84 EUR/m^3^ for *D. tertiolecta* and *C. sorokiniana*, respectively. This suggested that chitosan could be a valuable alternative flocculant for the large-scale cultures of auto-settling microalgae species including *T. striata*.

## Figures and Tables

**Table 1:** Aluminum and iron content (ppm), ash content (% DW), and water content (%) of fresh biomass from *T. striata*, *D. tertiolecta*, and *C. sorokiniana* harvested from the pilot-scale flocculation test assessing the most effective dose of each flocculant. Values are shown as mean ± standard deviation of three technical replicates. Values that were statistically different between the different samples of the same algae are marked with letters in the index: ^a^—different from untreated (control); ^b^—different from AlCl_3_-treated biomass; ^c^—different from FeCl_3_-treated biomass; and ^d^—different from chitosan-treated biomass. Letters indicate a significant difference (α = 0.05) between the different samples after Tukey or Dunn’s post hoc tests.

Element	Microalgae	Untreated (Control)	AlCl_3_ Treated	FeCl_3_ Treated	Chitosan Treated
Al (ppm)	*T. striata*	524.7 ± 17.8 ^b^	14,442.0 ± 2870.9 ^a,c,d^	562.7 ± 63.8 ^b^	673.0 ± 53.1 ^b^
	*D. tertiolecta*	182.3 ± 21.7 ^b^	34,335.7 ± 5041.1 ^a,c,d^	528.0 ± 43.3 ^b^	96.3 ± 6.7 ^b^
	*C. sorokiniana*	164.7 ± 21.4 ^b^	40,429.3 ± 585.7 ^a,c,d^	202.7 ± 11.8 ^b^	256.0 ± 10.1 ^b^
Fe (ppm)	*T. striata*	1219.7 ± 33.3 ^c^	1282.0 ± 162.8 ^c^	40,838.3 ± 2280.9 ^a,b,d^	1953.0 ± 68.8 ^c^
	*D. tertiolecta*	456.0 ± 47.1 ^c^	605.3 ± 41.0 ^c^	53,237.0 ± 4847.4 ^a,b,d^	766.3 ± 100.3 ^c^
	*C. sorokiniana*	1250.3 ± 75.1 ^c^	1368.3 ± 119.8 ^c^	59,462.0 ± 2339.3 ^a,b,d^	1515.3 ± 67.0 ^c^
Ash (%)	*T. striata*	18.8 ± 0.2 ^b,c^	23.1 ± 0.7 ^a,c,d^	27.3 ± 0.3 ^a,b,d^	19.0 ± 0.2 ^b,c^
	*D. tertiolecta*	11.9 ± 0.1 ^b,c^	26.4 ± 0.9 ^a,c,d^	35.4 ± 4.3 ^a,b,d^	18.9 ± 2.4 ^b,c^
	*C. sorokiniana*	7.2 ± 0.1 ^b,c^	20.6 ± 0.1 ^a,d^	29.4 ± 0.4 ^a,d^	8.6 ± 2.9 ^b,c^
Water content (%)	*T. striata*	70.3 ± 1.3 ^b,c,d^	81.3 ± 0.5 ^a,d^	80.6 ± 0.1 ^a^	78.2 ± 0.3 ^a,b^
	*D. tertiolecta*	75.6 ± 0.2 ^b,c,d^	82.4 ± 0.5 ^a^	82.0 ± 0.9 ^a^	82.8 ± 1.6 ^a^
	*C. sorokiniana*	76.8 ± 0.1 ^b,c,d^	85.9 ± 0.5 ^a,d^	87.1 ± 0.4 ^a,d^	89.2 ± 0.7 ^a,b,c^

**Table 2:** Cost of the harvesting process by traditional centrifugation and the two steps (centrifugation of the flocs obtained after flocculation using AlCl_3_, FeCl_3_, and chitosan) expressed in EUR/m^3^. All the flocculant prices were supplied by Spanish and European bulk suppliers.

Microalgae	Single-Step Centrifugation (EUR/m^3^)	Flocculation (EUR/m^3^)		
		AlCl_3_	FeCl_3_	Chitosan
*T. striata*	1.17	0.12	0.16	3.15
*D. tertiolecta*	1.17	0.14	0.24	7.86
*C. sorokiniana*	1.17	0.14	0.24	7.82
Estimated flocculant price (EUR/kg)	-	0.53	0.81	76.56

**Figure A3:**
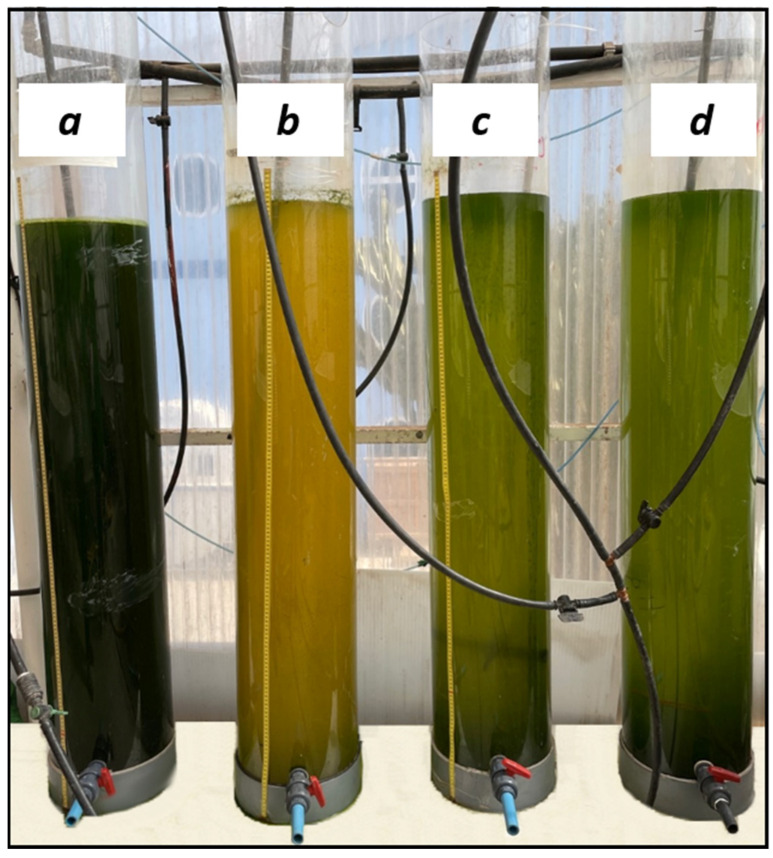
Color changes of the supernatant with different concentrations of FeCl_3_ at pilot-scale flocculation of *T. striata*: (**a**) Control culture, without flocculants, (**b**) 0.2 g/L FeCl_3_, (**c**) 0.1 g/L FeCl_3_, and (**d**) 0.08 g/L FeCl_3_.

## Data Availability

The original contributions presented in this study are included in the article. Further inquiries can be directed to the corresponding author.

## Appendix A

**Table A1:** Mineral (ppm), trace element (ppm), and heavy metal composition (ppb) in *T. striata* untreated and flocculant-treated biomass.

Elements		Untreated	AlCl_3_ Treated	FeCl_3_ Treated	Chitosan Treated
Mineral (ppm)	K	11,173.0 ± 175.7	9442.3 ± 144.8	10,512.7 ± 471.2	9675.7 ± 158.5
	Na	17,274.0 ± 673.7	32,002.0 ± 482.9	39,074.7 ± 495.9	22,005.7 ± 498.5
	P	3933.3 ± 152.8	3366.7 ± 152.8	3166.7 ± 251.7	4233.3 ± 513.2
	Mg	6752.7 ± 57.8	7676.0 ± 101.2	9548.3 ± 287.8	6456.0 ± 265.9
	Ca	23,605.7 ± 1042.2	8725.7 ± 853.5	9637.7 ± 211.6	13,003.3 ± 1812.4
Trace element (ppm)	Fe	1219.7 ± 33.3	1282.0 ± 162.8	40,838.3 ± 2280.9	1953.0 ± 68.8
	Al	524.7 ± 17.8	14,442.0 ± 2870.9	562.7 ± 63.8	673.0 ± 53.1
	Mn	64.3 ± 4.7	49.3 ± 7.6	46.3 ± 8.4	62.0 ± 6.6
	Cu	195.3 ± 6.0	183.0 ± 7.2	205.3 ± 14.0	230.3 ± 15.6
	Zn	184.3 ± 5.7	130.7 ± 2.5	120.7 ± 2.1	168.7 ± 3.2
Heavy metals (ppb)	Cr	29.3 ± 3.5	16.7 ± 1.5	113.0 ± 7.0	42.7 ± 5.0
	Pb	4.7 ± 0.6	10.0 ± 1.0	21.3 ± 2.1	18.7 ± 2.1
	Cd	4.0 ± 0.5	3.3 ± 0.2	0.0 ± 0.0	2.8 ± 0.2
	Co, Hg, As, Se	n.d. *	n.d.	n.d.	n.d.

* n.d.—not detected.

**Table A2:** Mineral (ppm), trace element (ppm), and heavy metal composition (ppb) in *D. tertiolecta* untreated and flocculant-treated biomass.

Elements		Control	AlCl_3_	FeCl_3_	Chitosan
Minerals (ppm)	K	9748.0 ± 112.2	5825.0 ± 301.7	4442.0 ± 199.3	8087.0 ± 454.6
	Na	22,823.7 ± 302.9	55,522.7 ± 684.2	35,855.7 ± 636.0	30,604.3 ± 529.3
	P	2800.0 ± 100.0	2633.3 ± 57.7	2500.0 ± 100.0	2400.0 ± 100.0
	Mg	3866.7 ± 81.8	10,903.3 ± 334.5	7746.0 ± 189.0	5612.7 ± 84.8
	Ca	866.0 ± 67.5	3198.0 ± 316.2	2670.0 ± 111.2	1145.3 ± 114.5
Trace elements (ppm)	Fe	456.0 ± 47.1	605.3 ± 41.0	53,237.0 ± 4847.4	766.3 ± 100.3
	Al	182.3 ± 21.7	34,335.7 ± 5041.1	528.0 ± 43.3	96.3 ± 6.7
	Mn	13.7 ± 1.5	15.0 ± 1.0	12.7 ± 2.1	30.3 ± 1.5
	Cu	202.0 ± 3.6	341.0 ± 32.2	330.3 ± 18.8	49.0 ± 2.6
	Zn	202.3 ± 8.1	213.3 ± 9.0	180.3 ± 6.0	62.7 ± 6.8
Heavy metals (ppb)	Cr	15.7 ± 0.6	21.3 ± 2.1	197.3 ± 11.0	55.7 ± 0.6
	Pb	5.2 ± 1.1	3.7 ± 0.5	20.0 ± 5.6	3.5 ± 1.0
	Cd	3.7 ± 0.5	1.0 ± 0.1	0.0 ± 0.0	0.0 ± 0.0
	Co, Hg, As, Se	n.d. *	n.d.	n.d.	n.d.

* n.d.—not detected.

**Table A3:** Mineral (ppm), trace element (ppm), and heavy metal composition (ppb) in *C. sorokiniana* untreated and flocculant-treated biomass.

Elements		Control	AlCl_3_	FeCl_3_	Chitosan
Minerals (ppm)	K	10,241.0 ± 1182.6	8775.0 ± 109.1	7658.0 ± 199.0	7732.3 ± 203.3
	Na	3914.0 ± 61.5	8541.3 ± 111.0	8757.0 ± 88.1	7767.3 ± 83.9
	P	9966.7 ± 208.2	19,633.3 ± 1021.4	18,300.0 ± 500.0	8400.0 ± 100.0
	Mg	3278.3 ± 83.1	3317.7 ± 36.6	3199.7 ± 93.4	3641.0 ± 54.7
	Ca	1417.7 ± 50.1	2851.0 ± 115.9	2210.0 ± 62.6	2076.0 ± 66.0
Trace elements (ppm)	Fe	1250.3 ± 75.1	1368.3 ± 119.8	59,462.0 ± 2339.3	1515.3 ± 67.0
	Al	164.7 ± 21.4	40,429.3 ± 585.7	202.7 ± 11.8	256.0 ± 10.1
	Mn	121.7 ± 3.1	114.3 ± 4.0	94.3 ± 5.0	118.0 ± 6.6
	Cu	20.0 ± 1.0	21.7 ± 2.5	29.7 ± 1.5	17.7 ± 1.5
	Zn	112.7 ± 12.0	110.7 ± 4.0	89.3 ± 3.1	120.0 ± 3.0
Heavy metals (ppb)	Cr	10.0 ± 1.0	7.7 ± 0.6	131.0 ± 17.1	18.3 ± 4.2
	Pb	1.8 ± 0.6	1.7 ± 0.4	2.2 ± 0.3	22.0 ± 1.0
	Cd	1.2 ± 0.2	2.2 ± 0.3	0.0 ± 0.0	1.6 ± 0.1
	Co, Hg, As, Se	n.d. *	n.d.	n.d.	n.d.

* n.d.—not detected.
